# miRNAs: The Key Regulator of COVID-19 Disease

**DOI:** 10.1155/2022/1645366

**Published:** 2022-10-29

**Authors:** Leyla Tahrani Hardin, Nan Xiao

**Affiliations:** Department of Biomedical Sciences at the Arthur A. Dugoni School of Dentistry, University of the Pacific, San Francisco, 94103 CA, USA

## Abstract

As many parts of the world continue to fight the innumerable waves of COVID-19 infection, SARS-CoV-2 continues to sculpt its antigenic determinants to enhance its virulence and evolvability. Several vaccines were developed and used around the world, and oral antiviral medications are being developed against SARS-CoV-2. However, studies showed that the virus is mutating in line with the antibody's neutralization escape; thus, new therapeutic alternatives are solicited. We hereby review the key role that miRNAs can play as epigenetic mediators of the cross-talk between SARS-CoV-2 and the host cells. The limitations resulting from the “virus intelligence” to escape and antagonize the host miRNAs as well as the possible mechanisms that could be used in the viral evasion strategies are discussed. Lastly, we suggest new therapeutic approaches based on viral miRNAs.

## 1. Introduction

SARS-CoV-2, which causes the severe acute respiratory syndrome COVID-19, has spread rapidly after it emerged at the end of 2019, and it has made a striking impact on world health and economy [1–5]. The World Health Organization (WHO) announced a public health emergency of international concern on 30 January 2020 and a global pandemic on 11 March 2020. Acute respiratory distress syndrome (ARDS) was shown to be the most common cause of death during the COVID-19 pandemic [4, 6, 7].

SARS-CoV-2 is a positive-sense-single-stranded RNA virus, and like other coronaviruses, SARS-CoV-2 has four structural proteins, known as the S (spike), E (envelope), M (membrane), and N (nucleocapsid) proteins. The N protein holds the RNA genome, and the S, E, and M proteins together constitute the viral envelope [8]. The cell entry is mediated by the viral glycoprotein (S) that contains the receptor-binding domain (RBD) that is required for binding to the host cell receptors and other domains that are required for membrane fusion and intracellular trafficking [9]. The human lung and intestine epithelial cells express abundant angiotensin-converting enzyme 2 (ACE2), which is assured as the receptor of SARS-CoV-2 [10]. ACE2 is mainly expressed in lung alveolar type 2, liver cholangiocyte, colon colonocytes, esophagus keratinocytes, rectum and stomach epithelial cells, and kidney proximal tubules [11]. The infectivity and ACE2 expression are gradually decreased from the upper airway to the lower airway [12].

After infecting the host cells, SARS-CoV-2 showed ability to launch a cytokine storm and to overcome the host immune system [13, 14]. Immunoregulatory agents seemed to play a key role in fighting against COVID-19. With more reports and ongoing research revealing the possible viral pathological pathways and virus-host interactions, mechanism of therapeutic strategies could be better elucidated, and alternative treatment are being investigated. Numerous vaccines were developed such as DNA vaccine, inactivated vaccine, viral vector vaccine, and RNA vaccine. However, it seems crucial to investigate the genetic drivers for SARS-CoV-2 and determine the humoral and cell-based players to establish long-term endurance [15].

Eukaryotes evolve noncoding molecules, miRNAs, to suppress unwanted genetic materials and transcripts [16, 17]. Circulating miRNAs can be secreted or produced as a result of diverse biological processes, such as (i) passive secretion from injured or damaged cell by chronic inflammation or apoptosis, (ii) active secretion via cell-derived membrane vesicles such as microparticles (exosomes), and (iii) active secretion by formation of protein-miRNA complex (e.g., HDL and Ago2) [18, 19]. miRNAs are endogenous small RNAs that contains 18 to 25 nucleotides which regulate protein translation through binding to complementary nucleotide of target mRNA, typically in the 3′-untranslated region (UTR). They are involved in posttranscriptional gene repression or degradation and can attach to multiple targets. miRNAs are recognized to participate in biological development, cell differentiation, apoptosis, and many other physiological and pathological processes and serve thus as watchdogs in the cell [20–24]. Previous studies have reported that miRNAs encoding sequences form up to 1% of the human genome [25].

These small molecules showed a promising therapeutic potential in many viral diseases such as herpes simplex virus (HSV), hepatitis C virus (HCV), human immunodeficiency virus (HIV), and influenza [26–31]. miRNAs constitute attractive candidates to be developed as vaccines and antivirals [32]. Vaccination can still be the fastest and most cost-efficient strategy to achieve comprehensive immunity [15].

This paper reviews the mechanisms that could be implicated in the host-virus miRNA interplay. In particular, we discuss the possible epigenetic control-based therapies through the cross-talk between the viral miRNAs and host machinery.

## 2. miRNAs: The Epigenetic Key Regulators against COVID-19

miRNAs are noncoding small molecules that are used in numerous therapeutic strategies for several diseases. They control the posttranslational phase of mRNAs through blocking the translation or through degradation. Studies showed miRNAs may inhibit, induce, or serve as enhancers of gene expression, which expand future therapeutic ideas and help reveal the biopathological mechanisms of many diseases [33–36]. It was proposed that miRNAs control cell cycle and gene expression through (i) a direct translation of the pri-miRNAs into regulatory short peptides named miRNA-peptides (MiPEP) which enhance the accumulation of their corresponding mature miRNAs [37], (ii) protein binding and structure changing [38], (iii) activation of TLRs [39], (vi) upregulating protein expression [40], (v) mitochondrial targeting [41, 42], (vi) regulation of other miRNAs [43], and (vii) upregulation of the gene transcription [44].

**Figure 1:**
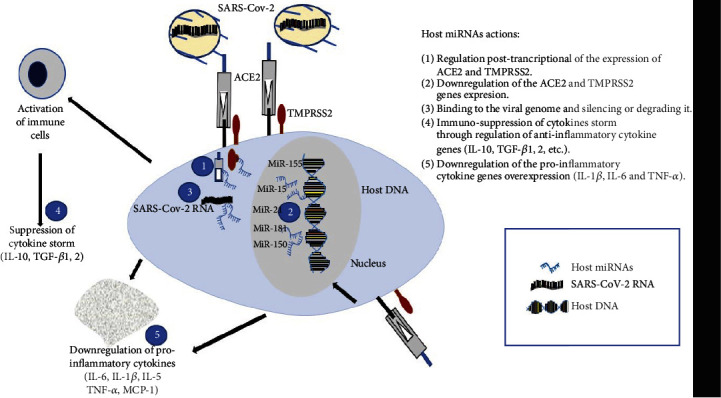
Plausible host miRNA action modes in SARS-CoV-2 infection. Host miRNAs may regulate the COVID-19 infection through 5 processes: 1—posttranscriptional regulation of the expression of ACE2 and TMPRSS2, 2—downregulation of the ACE2 and TMPRSS2 gene expression, 3—binding to the viral genome and silencing or degrading it, 4—immunosuppression of cytokine storm through regulation of anti-inflammatory cytokine genes (IL-10, TGF-*β*1,2, etc.), and 5—downregulation of the proinflammatory cytokine gene overexpression (IL-1*β*, IL-6, and TNF-*α*). Abbreviations: ACE2: angiotensin-converting enzyme; MiR: microRNA; TMPRSS2: transmembrane protease serine 2.

The interaction between miRNAs and viral RNA is the key to understand the pathological mechanisms of viral infections [45, 46]. Viruses are dependent on the host cellular machinery for their replication; at the same time, this makes them an easy target of the host destructors like miRNAs [30]. In the case of COVID-19 disease, recent studies indicate that miRNAs tune down SARS-CoV-2 infection probably through the following mechanisms (Figure 1).

1. Posttranscriptional regulation of the expression of ACE2 and TMPRSS2
2. Downregulation of the ACE2 and TMPRSS2 gene expression
3. Binding to the viral genome and silencing or degrading it
4. Immunosuppression of cytokine storm through regulation of anti-inflammatory cytokine genes
5. Downregulation of the proinflammatory cytokine gene overexpression

### 2.1. Posttranscriptional Regulation of the Expression of ACE2 and TMPRSS2

The angiotensin-converting enzyme 2 (ACE2) transmembrane protein on host cells plays a crucial role in SARS-CoV-2 infection as a spike glycoprotein receptor [47]. The spike protein is also cleaved and activated by cell surface transmembrane protease serine 2 (TMPRSS2) to facilitate membrane fusion and entry [48, 49]. ACE2 is expressed in multiple human tissues, with the highest expression in the gastrointestinal tract, kidney, testis, and heart [50].

miRNAs represent an attractive protein-targeted therapy, exceptionally for the proteins that cannot be targeted by other small molecules [51]. Several miRNAs such as miR-200b-3p, miR-200 c-3p, let-7c-5p, miR-98-5p, let-7 f-5p, miR-4500, and miR-27b may play a pivotal role in the deactivation of ACE2 or TMPRSS2 [22, 52]. miR-421 has also been previously identified as a potential ACE2 regulator [53]. A luciferase reporter system containing the 3′-UTR of ACE2 was exploited to identify the role of the miRNAs in ACE2 controlling; miR-421 and miR-483-3p showed to reduce ACE2 protein levels while the absence of the two miRNAs did not show to affect the ACE2 levels [53, 54]. According to Chen et al. [55], miR-421 and miR-483-3p controlled ACE2 expression through the translational mechanism rather the degradation of the transcripts. Also, the study of Kaur et al. [56] found that miR-214, miR-98, and miR-32 are able to repress TMPRSS2 with a high affinity to the binding sites on this protein especially miR-32.

### 2.2. Downregulation of the ACE2 and TMPRSS2 Gene Expression

Many miRNAs showed to regulate the ACE2 expression. The research of Hum et al. [57] showed a noticeable attention for miR-200 and miR-24 as they have been shown to correlate with the regulation of ACE2 and furin, respectively. Interestingly, studies highlighted that numerous miRNAs are involved in ACE2 expression such as miR-125b and miR-18, which directly target the 3′-UTR of ACE2 mRNA [58]. Sardar et al. [52] showed that considering the importance of cellular receptors, specifically ACE2, in SARS-CoV-2 infection, miR-27b regulates the ACE2 receptor. In another study, Chauhan et al. [22] reported that miR-200b-3p, miR-200 c-3p, and miR-429 could act against ACE2 and let-7c-5p, miR-98-5p, and miR-4500 may regulate TMPRSS2. Moreover, Widiasta et al. [58] reported that miR-18 upregulated the ACE2 expression in nephropathy. Along the same line, Khan et al. [59] identified six miRNAs that could regulate ACE2 in humans, namely, miR-362-5p, miR-421, miR-500a-5p, miR-500b-5p, miR-3909, and miR-4766-5p.

### 2.3. Binding to the Viral Genome and Silencing It or Provoking Its Degradation

Several studies showed that miRNAs binding on viral RNA could stop the virus replication, and these miRNAs have a significant role in gene regulation via binding to a specific region in 3′-untranslated region (3′-UTR) or open reading frame (ORF) to degrade mRNA or block the translation process [60]. Mature miRNAs specifically recognize complementary sequences found in the 3′-UTRs of target mRNAs and can facilitate translational repression and/or provoke mRNA degradation [61].

Arisan et al. [62] found that miR-8066 could block the SARS-CoV-2 N gene, which encodes a basic RNA-binding protein that acts as both structural and nonstructural protein. So, targeting this gene can reduce or repress the assembly and production of viral particles. Moreover, Nersisyan et al. [6] reported that six miRNAs including miR-21-3p, miR-195-5p, miR-16-5p, miR-3065-5p, miR-424-5p, and miR-421 are potentially regulating all human coronaviruses including SARS-CoV-2 through direct binding to the viral genome. miR-21-3p showed the highest binding level to the human coronavirus genome. miR-5047 inhibits the cleavage of the ORF1a/b polyprotein gene [63]. miR-1307-3p can also target human gene expression responsible for survival and proliferation (BCL2, PI3K pathway activators), clathrin-dependent endocytosis (AP2, PIP5K), and exocytosis (Actin) associated with virus cell entry and spread [64].

Chauhan et al. [22], through a bioinformatic analysis, determined miRNAs that target SARS-CoV-2 viral genes (e.g., repressing the spike protein gene) avoiding the viral binding on the host cell receptors. The imperfect attachment will result in an inhibition of the translation, while the perfect attachment will result in degradation of the mRNA [65]. In another bioinformatic study, Ivashchenko et al. [66] reported that miRNA-5197-3p can bind to SARS-CoV-2 genomic RNA and inhibit any gene expression modification in human cells. Along the same line, a computational study performed by Fulzele et al. [3] demonstrated that 873 human miRNAs were involved in binding and inhibiting SARS-CoV-2, and 558 of them targeted the virus genome. The highest binding score was observed with miR-15a-5p, miR-15b-5p, miR-30b-5p, miR-409-3p, miR-505-3p, and miR-548d-3p.

Ivashchenko and colleagues [66] identified human miRNAs that may alter the expression of SARS-CoV-2 genome. Their goal was to design a set of miRNAs that would specifically bind to the coronavirus genome to degrade it; they found miR-5197-3p endowed with the highest affinity to bind to SARS-CoV-2 genome. In addition, miRNAs can target virus genes and adjust their expression by degrading mRNA or deactivating the translation [3, 67]. Bertolazzi et al. [68] suggest that the miR-1207-5p family may bind to SARS-CoV-2 viral genome resulting in a deregulation of CSF-1, which may promote an inflammation in COVID-19 patients. According to Saçar Demirci and Adan [69], who investigated the plausible SARS-CoV-2 genes by host miRNAs, the results showed that except envelope (E) protein and ORF6, all viral genes (S, M, N, ORF1ab, ORF3a, ORF8, ORF7a, and ORF10) are targeted by multiple human miRNAs. An in silico study by Jafarinejad-Farsangi et al. [70] showed that miR-29 family of miRNAs was found to target 11 sites on SARS-CoV-2 genome. An experimental design consisted of luciferase reporter-plasmids carrying viral target sequences (pSpike) cotransfected with miRNA mimics in A549 cell line. The results showed an inhibition ranging from 60% (miR-30c and miR-15b) to more than 80% (miR-219a and miR-29a) [71].

### 2.4. Immunosuppression of Cytokine Storm through Regulation of Anti-Inflammatory Cytokine Genes

miRNAs have been shown to play a fundamental role in regulating the innate and adaptive immune response through several processes such as the maturation, secretion, and proliferation of T cells and B cells [72]. Nepotchatykh et al. [73] reported that miR-127-3p could regulate the expression of the BCL6 gene, resulting in a suppression of IL-10 expression, which has a crucial role in immune reaction alleviation. In addition, Yousefi et al. [74] discussed whether the host miRNAs can act either by regulating the host immune response, encoding for cytokine genes and changing the cell communication, or by directly targeting viral transcripts, degrading or silencing them. Schultz et al. [75] introduced more than two hundred miRNAs responsible for alleviating the cytokine exacerbation and three hundred miRNAs may at the same time inhibit the inflammatory agents and the cell death genes, hence preventing site injury. It has been shown that miRNAs play a crucial role in regulating the function of DCs, epithelial cells, monocytes, granulocytes, NK cells, and macrophages which are the principal actors in the innate immunity [76].

The analysis of differentially expressed genes targeted by host miRNAs demonstrated that 329 genes were upregulated and 452 genes were downregulated in the lung tissues from two postmortem men infected with SARS-CoV-2 compared to two healthy controls. Moreover, the results of enrichment analysis revealed that these genes were associated with the cytokine-mediated signaling pathway [70]. A previous study of Matsui et al. [77] showed that miRNAs can be translocated to the nucleus by importin 8 with AGO1 or AGO2; they determined that miR-589-AGO2 entered the nucleus to enhance the cyclooxygenase-2 (COX2) transcription. This mechanism could help explain how SARS-COV-2 is modulating the cytokine expression and open new therapeutic avenues.

A list of COVID-19-associated-miRNAs involved in immune response can be found in Table 1.

### 2.5. Downregulation of the Proinflammatory Cytokine Gene Overexpression

It was reported that in addition to the anti-inflammatory cytokines, the expression of proinflammatory cytokines is also controlled by miRNAs [78]. miRNA-155 increased in human cell lines infected with SARS-CoV-2 (Vero E6, Calu-3, and Caco-2) [79]. The serum concentration of miR-155 was significantly increased in COVID-19 patients compared to healthy controls [80]. This miRNA was associated with many viral infections [81, 82] and has been demonstrated to be an immune cell differentiation modulator [83]. Another study correlated the expression of miR-125b, miR-138, miR-199a, and miR-21 with the increase of TNF-*α*, IL-1*β*, and IL-6 [60]. In addition miR-146a and miR-146b were found to correlate with the increase of IL-8 in the acute respiratory distress syndrome and COPD [60, 84]. miR-323a-5p inhibits the translation of the ORF1a/b polyprotein gene [69] and miR-922 regulates PDCD1, which was found upregulated in SARS-CoV-2-infected samples and associated with T cell receptor signaling pathway, adaptive immune system regulation, T cell activation, leukocyte activation, and cell activation [85]. miR-326 showed correlation with leukocyte aggregation, defense response to fungus, neutrophil chemotaxis, granulocyte chemotaxis, neutrophil migration, regulation of immune system process, and cytokine-mediated signaling pathway [85, 86]. Five highly expressed miRNAs were identified in the placental MSC-EV, miR-92a-3p, miR-26a-5p, miR-23a-3p, miR-103a-3p, and miR-181a-5p, and showed to bind to the conserved sequence in SARS-CoV-2 genome and modulate the immune response [87].

On the other hand, Arisan et al. [62] found seven sequences in the SARS-CoV-2 mRNA that are highly similar to human miRNAs and they determined the probable molecular pathways associated with these miRNAs in the pathogenesis of COVID-19. Five of the seven miRNAs (miR-8066, miR-3934-3p, miR-1307-3p, miR-1468-5p, and miR-3691-3p) were implicated in the TGF-*β* signaling pathway. TGF-*β* is a mediator associated with lung development and homeostasis, and it also plays a role in the immunity, survival, migration, and apoptosis of host cells [88]. In addition, these miRNAs showed to be involved in viral entry and invasion, cytokine synthesis, and other disease complications such as pulmonary hypertension and cardiac fibrosis [89].

## 3. Ability of SARS-CoV-2 Escaping the Host Immune System through miRNAs

As discussed above, miRNAs are implicated in the host-pathogen interplay through silencing viral genes to fight against COVID-19. However, virus can develop new mechanisms to escape host antiviral response and to ensure its propagation [90]. Many studies reported that both virus and host encode miRNAs [91–95], and there are many types of viral miRNA-host mRNA interactions [30, 61]. Over the last two decades, hundreds of virus-encoded miRNAs have been identified [96]. Viral miRNAs aim to induce the degradation of host miRNAs, to inhibit or regulate their expression, and to target a large number of host genes involved in regulating cell proliferation, apoptosis, and host immunity [67, 97–99, 99].

**Figure 2:**
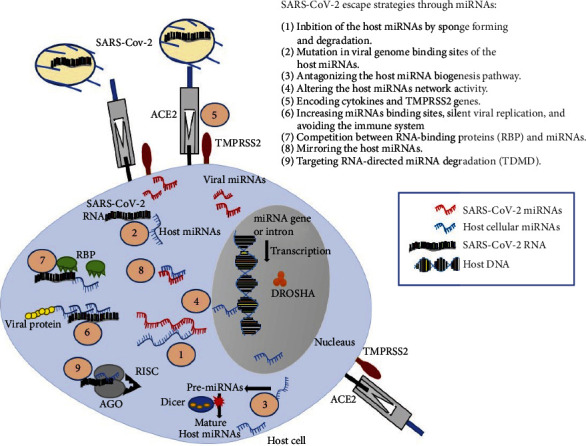
Probable SARS-CoV-2 escape strategies through miRNAs. SARS-CoV-2 can escape the immune response through strategies: 1—inhibition of the host miRNAs by sponge forming and degradation, 2—mutation in viral genome binding sites of the host miRNAs, 3—antagonizing the host miRNA biogenesis pathway, 4—altering the host miRNA network activity, 5—encoding cytokines and TMPRSS2 genes, 6—increasing miRNA binding sites, silent viral replication, and avoiding the immune system, 7—competition between RNA-binding proteins (RBP) and miRNAs, 8—mirroring the host miRNAs, and 9—targeting RNA-directed miRNA degradation (TDMD). Abbreviations: ACE2: angiotensin-converting enzyme; TMPRSS2: transmembrane protease serine 2; AGO: argonaute; Dicer; RBP: RNA-binding protein; RISC: RNA-induced silencing complex.

The latest research indicated that SARS-CoV-2 could escape the attack of host immune system through miRNAs as well. The probable mechanisms are (Figure 2) as follows:

1. Inhibition of the host miRNAs by sponge forming and degradation
2. Mutation in viral genome binding sites of the host miRNAs
3. Antagonizing the host miRNA biogenesis pathway
4. Altering the host miRNA network activity
5. Encoding cytokines and TMPRSS2 genes
6. Increasing host miRNA binding sites, silencing viral replication, and avoiding the immune system
7. Competition between RNA-binding proteins (RBP) and host miRNAs
8. Mirroring the host miRNAs
9. Targeting the RNA-directed miRNA degradation (TDMD) pathway

### 3.1. Inhibition of the Host miRNAs by Sponge Forming and Degradation

SARS-CoV-2 mRNAs could regulate host miRNA levels by acting as sponges to evade the immune system and to promote the viral cycle. The high potential miRNA binding sites could offer a very effective defense by reducing the cellular miRNA levels during the infection [1]. The viral sequence, once expressed, can interact with the host's miRNA regulatory machine by sequestering the selected miRNAs.

It is reported that the high concentration of viral RNA in the cell may sequestrate miR-1207-5p therefore contributing to CSF1 release leading to enhanced macrophage recruitment and activation [68]. Another study reported that there are many miR-16 binding sites in human coronavirus HCoV genomes, including SARS-CoV-2. The viral RNA may act as a “sponge” to sequester miR-16 [57], consequently decreasing its abundance in infected cells resulting in a decrease of its targets in the host [100]. miR-16 is known by its role in trigging apoptosis via downregulating survival factor BCL2 [101].

Along the same line, a total of 14 high-confidence mature miRNAs, which are strongly likely to bind with the SARS-CoV-2 genome and are expressed in diverse respiratory epithelial and immune cells, were identified and some researchers suggest that there is a possibility that the SARS-CoV-2 genome may sequester cellular miRNAs during pathogenesis, especially in high copy numbers in infected cells [102]. Moreover, the study of the potential role of the 28 miRNAs, which were uniquely specific for SARS-CoV-2, showed that the virus by its potential reduction of the host's miRNA pool may modulate specific gene expression to suppress immunity or prevent activation of unfolded protein response- (UPR-) related apoptosis and promote infected cell survival and thus continue its replication cycle [1]. A study using bioinformatic approaches showed that the SARS-CoV-2 virus can serve as a “sponge” for human miRNAs such as miR-374a-5p, let-7f-1-3p, miR-374a-3p, miR-548d-3p, and miR-23b-3p [103]. miR-302c-5p, which is expected to be sponged by the SARS-CoV-2 genome, can potentially lead to increased ACE2 expression which has already been reported to be associated with severe COVID-19 cases [103, 104].

### 3.2. Mutation in Viral Genome Binding Sites of the Host miRNAs

Another way used by SARS-CoV-2 to evade the immune system and to propagate is mutation. It was shown that mutation in RNA viruses is quite frequent because of the RNA-dependent RNA polymerase's inadequate proofreading activity [105, 106]. SARS-CoV-2 could change the binding sites of host miRNAs through mutations. The pathogenicity of the virus can be deeply affected by mutations, e.g., through modification of the RNA secondary structure or creating new binding sites for host miRNAs [107].

A study of Hosseini Rad SM and McLellan [108] identified a lost binding site for miR-197-5p, which was located in the nonstructural protein 3 (Nsp3) sequence. NSP3 is the largest protein encoded by the coronavirus genome and plays an essential role in replication and transcription. The Nsp3 synonymous C3037U mutation could impact viral fitness. Van Dorp et al. [109] demonstrated that the Nsp3 C3037U mutation was significantly associated with virus transmission. miR-197-5p is overexpressed in patients with cardiovascular disease that demonstrate an increased susceptibility to SARS-CoV-2 infection [110]. A number of researches showed that miR-197-5p was associated with defense mechanisms against certain viruses such as HBV, HCV, and H7N9 [111–113]. The loss of miR-197-5p-mediated defense against SARS-CoV-2 may be related to the increased mortality noted in this patient group [7].

Chan et al. [114] reported that mutations in SARS-CoV-2 3′-UTR lead to virus escape from the host immune system. In the study of Liu et al. [107], both miR-939-5p and miR-146b-3p, which were involved in maintaining homeostasis by decreasing inflammatory response, were predicted to be incapable of binding to the mutant-type genome. Park et al. [115] found that most sites of the viral genome that bind to 3′-UTR miRNAs are conserved and rarely mutated, whereas the binding sites of miR-181a-5p, miR-92a-3p, miR-92b-3p, and miR-25-3p were mutated in the SARS-CoV-2 genome from Australian samples.

### 3.3. Antagonizing the Host miRNA Biogenesis Pathway

Several viruses showed to hijack the host miRNA biosynthesis through viral proteins such as Ebola's VP35 [116] and HCV's core protein [117]. There is possibility that SARS-CoV-2 could use the same evasion strategy. Many RNA viruses showed to inhibit the maturation of the host miRNAs through argonaute (AGO) effectors [91]. The 2b protein of the cucumber mosaic virus (CMV) appeared to be a repression factor of AGO1 slicer function [118], while the p19 protein of the tomato bushy stunt virus (TBSV) showed to impede the activity of RNA-induced silencing complex (RISC) loading activity through binding to small double-stranded RNAs [119]. It was reported that Dicer, which represents the most critical regulator of the biogenesis of miRNA and small interfering RNA [120], could be sequestrated by the noncoding (nc) subgenomic flavivirus RNA [121]. According to Arora et al. [122], the receptors of Ddx58, which contributes to the miRNA biogenesis and mRNA splicing, showed to be highly upregulated in COVID-19 disease. SARS-CoV-2 hijacks Ddx58 to foster its replication [60, 122]. Bartoszewski et al. [1] identified 10 pre-miRNA sequences in the SARS-CoV-2 RNA sequence that could potentially interfere the human miRNA biogenesis, hypothesizing that maybe SARS-CoV-2 encode in its own pre-miRNA sequences that could mature in human cells. Arisan et al. [62] found in their predictive study that SARS-CoV-2 genome reveals RNA structures (miRNAs), which could be conceivable Drosha and Dicer substrates. However, another RNA virus, the influenza virus, replicates inside the host nucleus and utilizes the nuclear miRNA processing factor Drosha to express viral miRNAs [67, 123]. So, the RNA molecules are accessible to the miRNA biogenesis machinery in the nuclei. SARS-CoV-2 could use this alternative to synthesize its viral miRNAs.

### 3.4. Altering the Host miRNA Network Activity

Viruses have been shown to change the host miRNA expression landscape, and they are able to bind directly to host miRNAs or to use them for their needs to stabilize genome, to propagate, and to maintain diseases [46, 57, 61].

The host miRNAs can be dual hatted and can sometimes participate in the viral escape by modifying host immune mediators [91]. Khan et al. [59] reported that during SARS-CoV-2 infection host miRNAs may downregulate the signaling of different TLRs, which play a fundamental stimulatory role in inducing the host antiviral activity (i.e., secretion of interferons and other inflammatory cytokines). A recent *in silico* study showed that six host miRNAs (hsa-miR-32-5p, hsa-miR-98-3p, hsa-miR-214-3p, hsa-miR-421, hsa-miR-423-3p, and hsa-miR-1246) can target ACE2 and TMPRSS2 and the number of binding sites of hsa-miR-98-3p and hsa-miR-423-3p was the highest (a match of 6 nucleotides) [124]. These miRNAs were found to increase in critical COVID-19 patients. In addition, a functional analysis showed that the target genes are involved in the viral process, including viral RNA replication and viral entry into the host nucleus or regulation of the viral genome replication [124].

In the study of Saçar Demirci and Adan [69], a total of 1367 human genes seem to be targeted by SARS-CoV-2 viral miRNAs. Some of the predicted targets of these viral miRNAs in human genes have roles in transcription. The eukaryotic transcription regulators would be the most important targets of 18 SARS-CoV-2-derived mature miRNAs; these targets are all involved in transcription process [69]. Mostly viral miRNAs have shown their role in overall pathogenesis by regulating host genes [125]. The findings of an in silico study have shown an analogy between the SARS CoV2 genome and regulatory host miRNA such as miR8066, miR5197, miR3611, miR3934-3p, miR1307-3p, miR3691-3p, and miR1468-5p [59].

SARS-CoV-2 hijacks the miRNA expression for an immunomodulatory activity; the virus provokes the cytokine storm to escape the immune system and upregulate or downregulate the miRNA expression, which lead to maladaptive immune system while maintaining active replication [126, 127]. It was found that 35 miRNAs were upregulated and 38 miRNAs were downregulated in COVID-19 patients compared to healthy donor. Notably, miR-6501-5p and miR-618 expression levels were 1.5-fold higher and miR-627-5p 2.3-fold lower in COVID-19 patients than in healthy donors. The expression of miR-183-5p, miR-627-5p, and miR-144-3p was also reduced [128]. Another study reported that 55 miRNAs were differentially expressed in the plasma of COVID-19 patients compared to healthy controls. 20 miRNAs were upregulated and 30 were downregulated. The most strongly upregulated miRNA was miR-31-5p, which was reported as an important factor in inflammatory disorders [129, 130], while the most downregulated miRNAs were miR-1275, miR-3617-5p, and miR-500b-3p [131]. The associated transcription factors of miRNAs miR-429 and miR-1286 were upregulated, and their target genes were downregulated, suggesting they might have some roles in this host-virus interactions [132]. In an in silico-based study, miRNAs were shown to act in synergy, and it was demonstrated that SARS-CoV-2 can alter the miRNA network activity and reroute it for its advantage [74]. The dysregulation of miR-1207-5p-target genes during SARS-CoV-2 infection may lead to uncontrolled inflammation in most severe COVID-19 cases [68]. Several other researches showed that miR-1207-5p is involved in contributing to virus propagation and especially to the virus reviving from latency phase [133]. Moreover, Arisan et al. detected mutations in miR-1307 and miR-8066, miR-129-2-3p, and miR-129-2-3p. These three miRNAs are potentially involved in pathways related to SARS-CoV-2 infection and host defense such as mucin type O-glycan biosynthesis, TGF-signaling pathway, amphetamine addiction, cytokine-cytokine receptor interaction, and nicotinate-nicotinamide metabolism [62].

Based on the available evidence, it is reasonable to hypothesize that SARS-CoV-2 may change the host's miRNAome to create a proviral microenvironment for its own replication and propagation, through modifying host miRNAs and consequently altering cellular machineries during infection. Moreover, the viral-derived miRNAs are one of many options that SARS-CoV-2 uses to escape the host immune system through interfering host gene expression, blocking or inducing host miRNAs.

### 3.5. SARS-CoV-2 miRNAs and Proteins Are Involved in Encoding Cytokines and TMPRSS2 Genes

A computational study performed by Liu et al. [134] showed that SARS-CoV-2-derived miR-147-3p potentially could enhance the expression of TMPRSS2, which is an entry ACE2 receptor enhancer, with the function of strengthening SARS-CoV-2 infection in the gut. Moreover, Liu et al. [134] reported that 11 SARS-CoV-2-derived miRNAs were found to be bound to the 5′-UTR of 13 target genes, including the binding between a virus-encoded miRNA MR385-3p and TGFBR3 (transforming growth factor beta receptor 3), which is known to be implicated in the innate and adaptive immune response [135]. Gene ontology (GO) and pathway functional enrichment of the targeted genes by the SARS-CoV-2 miRNAs revealed different pathways implicated in several immune signaling, such as T cell-mediated immunity, autophagy, TGF-*β* signaling, mTOR signaling, and TNF-*α* signaling which are particularly targeted by SARS-CoV-2 [59]. Six SARS-CoV-2 miRNAs (SARS-CoV-mir-1-5p, SARS-CoV-mir-2-5p, SARS-CoV-mir-3-5p, SARS-CoV-mir-4-5p, SARS-CoV-mir-5-5p, and SARS-CoV-mir-6-5p) were found to target immune-related genes that play an important role in immune pathways like TNF and chemokine signaling pathways [136]. Another study determined that miR-8066, 5197-3p, 4778-3p, and 6864-5p sequence is found on SARS-CoV-2 genomes. These SARS-CoV-2-mediated alterations of miRNAs may act as autocrine or paracrine agonists of host cells to trigger proinflammatory cytokines, due to their increased NF-KB activity [62].

Virus-encoded proteins can inhibit the upregulation of host miRNAs which are triggered upon viral infection to suppress the innate immune response. Zhai et al. [137] identified a Borna virus-encoded protein which is able to suppress the expression of miR-155, which is implicated in innate immune responses. The suppression of miR-155 showed to attenuate lung cytokine storm in mice infected with SARS-CoV-2 [138, 139]; here, we suggest that miR-155 could be regulated by viral proteins as well. Along the same line, miR-146a, miR-21, and miR-142 were found in COVID-19 patients triggering the inflammatory machinery through activating MAPK and NF-Ƙb signaling [140]. It was identified that restricting miR-21 or miR-142 would promote multiple cascading reactions and activation of the JAK-STAT cell signaling circuit, resulting in proinflammatory status advancements [125]. Trobaugh and Klimstra [46] suggested that viral protein-mediated changes in miRNA expression may occur through an indirect mechanism governed by viral inhibition of the innate immune response rather than a direct interaction between the viral protein and the transcription factors that regulate miRNA expression.

### 3.6. Increasing Host miRNA Binding Sites, Silencing Viral Replication, and Avoiding the Immune System

Nersisyan et al. [6] reported that host miRNAs had dozens of binding regions on SARS-CoV-2. miR-16-5p, miR-195-5p, and miR-424-5p viral binding sites showed to be highly conserved across human coronaviruses and their strains. Along the same line, miR-29 family (miR-29a, miR-29b, and miR-29c) was shown to have the most binding sites (11 sites) on SARS-CoV-2 and eight binding sites for miR-29a-3p, miR-29c-3p, miR-21-3p, miR-761, and miR-3130-3p [70]. Increasing the host miRNA binding sites could be explained by the fact that SARS-CoV-2 shuts down its own viral cycle rate for evading perception and destruction by the host immune system [141]. Such behavior was highlighted, for example, in the case of eastern equine encephalitis virus (EEEV); miR-142-3p binds to EEEV genome and represses the viral translation and replication only in myeloid cells likely limiting both innate and acquired immune responses to EEEV [142]. Also, a previous study showed that the viral miR-BART22, which is encoded by Epstein-Barr virus (EBV), modulates the latent membrane protein 2A (LMP2A) expression. This may facilitate the escape of EBV-infected cells from host immune surveillance [143].

Similarly, SARS-CoV-2 showed to alter the circulating levels of miR-21 and miR-16, which are known to suppress the secretion and expression of TNF-*α* and IL-6 mRNA, activation of macrophages, and proliferation of smooth muscle cells in normal cellular environment [63, 144, 145]. Bautista-Becerril et al. [145]explained this by the binding of these miRNAs to the virus receptors or its target mRNAs. Although miRNAs canonically serve as posttranscriptional repressors of cellular mRNAs, they may have unconventional roles as activators as, for example, in the case of the miR-122 : HCV relationship. A possible explanation is that miR-122 binds to the SARS-CoV-2 RNA genome in the same way that it binds with the HCV genome, and the host-virus RNA-RNA interactions ease viral cycle. There is possibility that similar mechanism might apply in COVID-19 progression during which some host miRNAs turn out to be a positive cofactor of SARS-CoV-2.

### 3.7. Competition between RNA-Binding Proteins (RBP) and miRNAs

In addition, the 50 and 30 untranslated regions (UTRs) of the SARS-CoV-2 RNA contain binding sites for cellular miRNAs and RNA-binding proteins (RBPs) [89]. The host miRNAs target the viral RNA, generally leading to RNA degradation, and the translation of viral proteins can be induced by the binding of RNA-binding proteins (RBPs) such as CUG-binding protein (CUG-BP) and transactive response DNA-binding protein (TARDBP) [146]. For example, in a SARS-CoV-2 variant, a mutation happened in the binding site for CUG-BP and turned it into a TARDBP binding site via a change of “C” to “U” on position 241; consequently, viral infectivity and mortality increased [147]. Mukherjee and Goswami [146] reported miR-34b-5p bond RNA-binding motif single-stranded-interacting protein 3 (RBMS3) at binding sites for host miRNA to stabilize this RBP, avoiding miRNA strike and RISC-induced RNA decomposition. In addition, miRNA binding might be prevented by a specific nucleotide modification in an overlapping binding site, while RBP binding remains possible; an example of this scenario is seen in miR-9-5p and HNRNPA1 [146].

RNA-protein interactions showed to block regions of the virus genome, making them unreachable for host cellular miRNA targeting, creating a competitive site binding between miRNAs and RNA-binding proteins [102, 148]. We suggest the presence of the same mechanism for SARS-CoV-2 involving the virus-derived miRNAs and RBP-binding proteins.

### 3.8. Viral miRNAs Mirror the Host miRNAs

SARS-CoV-2 showed to secrete identical miRNAs to the human miRNAs. Another mechanism that could be implicated in the viral escape from the regulation of host miRNAs is mirroring the host army. According to Henzinger et al. [89], SARS-CoV-2 could mirror host miRNAs through the expression of identical viral miRNAs.

It has been reported that virus-encoded miRNAs share seed region, which is the binding determinant region [149], with host miRNAs, and this was observed, at least, for three viruses: Kaposi's sarcoma-associated herpesvirus (KSHV), Marek's disease virus 1 (MDV1), and bovine leukemia virus (BLV). These viral miRNAs have been shown to negatively regulate transcripts via the same target docking sites as their counterpart host miRNAs [150]. Mimicking a host miRNA allows a viral miRNA to potentially regulate hundreds of transcripts that have evolved target sites for a particular host miRNA, as the case of the seed sharing between the KSHV-encoded miR-K11 and human miR-155 which was associated with the downregulation of a common set of host mRNA transcripts [151, 152]. miR-155 showed to have an oncogenic role in many diseases [153–155]; also, it showed to play a pivotal role in the immune response [156–160]. Moreover, miR-155 appeared to play a role in the cell differentiation of B cells, T cells, and macrophages [93]. Many studies reported the high miR-155 expression in COVID-19 patients [79, 138, 139]. SARS-CoV-2 might also evolve mimics of host miRNAs to attain the memory B and T cell populations found during a latent infection.

### 3.9. Target RNA-Directed miRNA Degradation (TDMD) Pathway

Another phenomenon observed for the coronavirus's infection is the TDMD, where the repression of the host miRNAs does not engage the miRNA gene transcription or the miRNA biogenesis; however, the mature miRNAs, after they are loaded into argonaute (AGO), bind to a highly complementary viral RNA region by a mechanism involving nucleotide addition, tailing, trimming, and decay [161–163]. The computational study of human miRNAs and coronavirus RNA interaction of Nersisyan et al. [141] showed that the expression of miR-21-3p in mouse lungs increased 8-fold upon SARS-CoV infection, while miR-21-5p (the “guide” strand of the same pre-miRNA hairpin) exhibited only 3-fold increase. They hypothesized that the unsimilar upregulation of miR-21 arms could be due to SARS-CoV-2 RNA triggering of the host miRNA degradation. miR-21-3p was previously implicated in several RNA virus infections such as influenza A virus [164], Chandipura virus [165], and Coxsackievirus [166].

## 4. Viral miRNAs as Therapeutic Approaches

RNA viruses have been showed high mutation rate which is a million times higher than the host mutation rate [167]. Considering that coronaviruses are rapidly evolving in humans, this enables them to escape therapeutic therapies and vaccine-induced immunity [168]. A new study demonstrated that SARS-CoV-2 receptor-binding domain (RBD) can tolerate large numbers of simultaneous mutations and evade antibody neutralization through an N-linked glycan [169]. With this continuous fast viral evolution, it will not be surprising that highly antibody-resistant strain emerges.

Most of the previous studies were focusing on mRNAs and viral proteins as therapeutic alternatives or as vaccine models. Based on the crucial role that miRNAs can play in COVID-19 infection, using miRNAs as targets may represent the dawn of a new therapeutic era. Compared to targeting host miRNAs, which are involved in much more complexed physiological pathways, targeting viral miRNAs would be more achievable.

**Figure 3:**
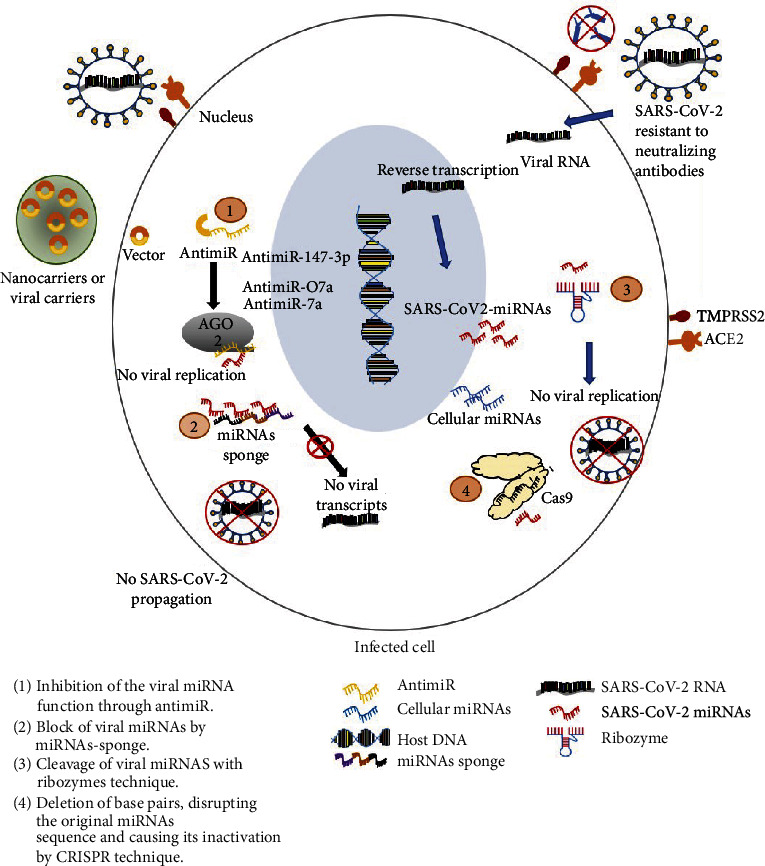
Targeting viral miRNAs for therapeutic avenues. 1—inhibition of the viral miRNA function through antimiR; 2—blocking of viral miRNAs by miRNA sponge; 3—cleavage of viral miRNAs with ribozyme technique; 4—deletion of base pairs, disrupting the original miRNA sequence and causing its inactivation by CRISPR technique. Abbreviations: ACE2: angiotensin-converting enzyme; AGO2: argonaute 2; CRISPR: clustered regularly interspaced short palindromic repeats; TMPRSS2: transmembrane protease serine 2.

Like their hosts, most pathogens, such as viruses, also encode miRNAs to promote their survival [127, 170]. Mature viral miRNAs can be targeted using antisense oligonucleotides, known as antimiRs [171], miRNA sponges, ribozymes, and CRISPR technique. Some probable mechanisms are (Figure 3) as follows:

1. Inhibition of the viral miRNA function through antimiR
2. Blocking multiple viral miRNAs through miRNA sponge
3. Cleavage of viral miRNAs with ribozyme technique
4. Deletion of base pairs, disrupting the original miRNA sequence, and causing its inactivation by CRISPR technique

A miRNA antagonist is usually introduced as a chemically modified miRNA which is known as antimiR or antagomiR that could complementarily bind to the mature miRNA strand. Thus far, scientists developed various chemical structures of antimiR oligonucleotides with the goal to maximize their binding affinity, stability, and *in vivo* delivery [172]. A complementary strand to a viral miRNA could be constructed based on the highly conserved viral miRNA sequence, inserted to a vector with a sequence of RNA-binding proteins to provoke viral miRNA degradation. Several antimiRs were developed as treatments for viral diseases; miravirsen, which targets miR-122, was used for treatment of hepatitis C virus to reduce liver and plasma viral titers [173], and anti-EBV-miR-BART7-3p inhibits the tumorigenicity of Epstein-Barr virus (EBV) in mouse infected cells [174].

miRNA sponges are plasmid constructs which use a transgenic overexpression of RNA molecules containing multiple high-affinity miRNA complementary binding sites to a miRNA of interest to block the function of a given miRNA or a miRNA family [100]. These transcripts can efficiently sequester specific miRNAs, preventing their binding to endogenous target genes [175]. RNA oligonucleotides containing many viral miRNA binding sites could be used as a fish trap in order to sequester them.

Moreover, the viral miRNAs could be modulated through synthetic ribozymes, which target the highly conserved regions in viral miRNAs implicated in virus replication, assembly, and release processes. It is possible to exploit the site-specific cleavage ability of hammerhead ribozymes to design synthetic ribozymes and establish that they can cleave viral miRNAs [176].

Along the same line, many studies demonstrated that cloning CRISPR/cas9 constructs with single-guide RNAs specifically targeting biogenesis processing sites of selected miRNAs can robustly and specifically reduce the expression of these miRNAs [177–179]. The CRISPR/cas9 technology showed be effective in modifying miRNA precursor structure and disrupting its processing [180].

Several viral miRNAs were identified by recent studies, and they interestingly represent therapeutic candidates particularly those playing roles in viral replication.

There have been reports on viral miRNAs that could be the potential targets. miR-7a is a viral miRNA which hijacks the host miRNA machinery and antagonize host miRNAs to repress the expression of interferon-stimulated genes (ISGs) through sequence complementarity to sites located in their 3′-UTR [181]. Along the same line, miR-O7a is derived from the beginning of the coding sequence of the ORF7a transcript, which is the most abundant subgenomic viral RNA detected during SARS-CoV-2 infection [79]. Indeed, it was demonstrated that CoV2-miR-O7a associates with human AGO proteins and that it can repress human targets [182]. Also, miR-nsp3-3p, a viral miRNA which was recently identified by Fu et al., exhibits comparable sensitivity, specificity, and precision to that of multiomics and could be used as a biomarker or a therapeutic target [183]. Liu et al. [107] found that a virus-encoded miRNA mir-147-3p could enhance the expression of TMPRSS2 with the function of strengthening SARS-CoV-2 infection in the gut; an antimiR could be constructed to inhibit the TMPRSS2 overexpression. Moreover, developing nanoformulations of the COVID-19-related miRNAs can overcome delivery limitations [60].

## 5. Conclusion and Future Perspectives

In this article, we reviewed the role of host and viral miRNAs during viral infection and disease development. It was reported that SARS-CoV-2 infection relies on epigenetic modulators of the host cell and the immune actors. We also suggested potential molecular mechanisms of miRNA therapy in treating COVID-19 disease based on published studies.

The study of microRNAome could help identify biomarkers of COVID-19, understand the viral infection and disease progression mechanisms, develop curative alternatives, and possibly help to relieve the long-haul symptoms in patients that recovered from SARS-CoV-2 infection. Targeting the host cellular miRNAs is challenging, and any modification may lead to side effects on organism, as these molecules have multigenic impact. However, targeting the highly conserved viral miRNAs seems to be an appropriate druggable target especially now when SARS-CoV-2 continue mutating. Although the biogenesis, mechanisms, and roles of viral miRNAs are not well determined, a considerable advancement has been achieved due to the computational analysis and sequencing techniques. Future investigations will provide more evidence on miRNA approaches to fight COVID-19 and possibly other virus infection.

## Figures and Tables

**Table 1:** COVID-19-associated miRNAs involved in immune response.

miRNAs	Role in the immune response	miRNA expression	References
miR-15a-5p	Binding to SARS-CoV-2-encoded transcripts Involved in immunity enhancing	Upregulated	[89, 184, 185]

miR-15b	Positively correlated with inflammation Regulating the host immune response	Upregulated	[140, 186, 187]

miR-17-5p	Having actual target sites in SARS-CoV-2 and in inflammation response, role not well known	Downregulated	[3]

miR-21	Regulating inflammatory response Correlated with macrophage numbers and with MCP-1 protein expression in in the bronchoalveolar lavage (BAL) supernatant 27 binding sites on SARS-CoV-2 genome with the highest target scores	Upregulated	[80, 141, 188, 189]

miR-23a-3p	Binding to the complemented sequence which led to the recovery of the cytopathic effects Related to cytokine–cytokine receptor interactions, TNF, NF-*κ*B, chemokines, Toll-like receptors, and the Jak–STAT signaling pathway	Not reported	[87]

miR-25-3p	Having strong binding potential against TMPRSS2 in COVID-19 disease Implicated in the SARS-CoV-2 talk with host cell Downregulated in COPD	Downregulated	[74, 190–193]

miR-29a	Identified in COVID-19 infection Anti-inflammatory in cancer and other diseases	Upregulated	[70, 139, 194]

miR-31-5p	Mediating inflammatory signaling	Downregulated	[129, 131, 140, 195]

miR-98	Negatively correlated with inflammatory cytokines	Downregulated	[196, 197]

miR-99a-5p	Targeting proinflammatory genes	Downregulated	[140, 198]

miR-124	Targeting the 3′-UTR binding sites on IL-6 gene in COVID-19 disease Suppressing LPS-induced p65 expression and cell apoptosis Decreasing the levels of inflammation and pulmonary injury	Not reported	[199, 200]

miR-125b	Binding to the 3′-UTR region of TNF and IFN and also to the 3′-UTR of factor XIII gene	Downregulated	[75, 193]

miR-142-3p, miR-142-5p	Promoting inflammatory process Downregulated in COVID-19 infection	Downregulated	[140, 188]

miR-146a-5p	Acting as biomarker and providing a molecular link between inflammation and the COVID-19 infection	Downregulated	[140, 201, 202]

miR-149-3p	Suppressing hepatic inflammatory response through antagonizing the STAT3 signaling pathway	Not reported	[1]

miR-150	Predicted to have binding sites in the SARS-CoV-2 genome Alleviated LPS-induced inflammatory response and apoptosis A target in the development of anti-inflammation and antiapoptosis drugs for sepsis treatment	Downregulated	[3, 63, 69, 160, 193, 203]

miR-155	Reducing proinflammatory cytokines Increased antiviral and anti-inflammatory cytokine responses in the lungs	Upregulated	[63, 138]

miR-181a	Inhibiting secretion of proinflammatory factors especially the increase in TNF-*α*	Downregulated	[140, 204]

miR-195	Promoting resolution of inflammation	Upregulated	[131, 196, 205, 206]

miR-200 family	Members of this family are proinflammatory as well as anti-inflammatory Regulating ACE2 (virus entry) in respiratory cells in COVID-19 disease	Upregulated	[207–209]

miR-320 family	Significantly correlated with CRP, IL-6, and D-dimer levels	Downregulated	[210]

miR-342-5p	Targeting N (nucleocapsid) protein gene in SARS-CoV-2 infection Promoting inflammatory activation of macrophages		[69, 211]

miR-421	Aggravating inflammatory response in lung tissues Identified as an ACE2 regulator Targeting sites on 3′-UTR of ACE2 in human genome	Downregulated	[53, 57, 212–215]

miR-769-3p	Attenuating the cell death, avoiding tissue damage by targeting synergistically the 3′-UTR region of the TNF and IFN genes inhibiting their protein translation	Not reported	[75]

miR-1207	Derepression of its target CSF1 results in acute inflammatory response in COVID-19	Not reported	[68, 68, 213]

miR-1307	Promoting inflammatory responses Involved in TGF-*β* signaling, inflammatory response, oxygen dependency, persistent wheezing, and chronic lung diseases	Downregulated	[62, 64, 213, 216]

miR-let-7	Related to inflammatory responses in COVID-19 disease especially miR-let-7b-5p and miR-let7a-5p	Downregulated	[63, 215]

Abbreviations: ACE2: angiotensin-converting enzyme 2; BAL: bronchoalveolar lavage; COPD: chronic obstructive pulmonary disease; LPS: lipopolysaccharides; TMPRSS2: transmembrane protease serine 2; STAT: signal transducer and activator of transcription 3; UTR: untranslated region.

## Abbreviations

ACE2: Angiotensin-converting enzyme 2
AGO: Argonaute
cDNA: Complementary DNA
COPD: Chronic obstructive pulmonary disease
COVID-19: Coronavirus disease 2019
CSF1: Macrophage colony-stimulating factor
CRISPR: Clustered regularly interspaced short palindromic repeats
IL: Interleukin
MERS-CoV: Middle East respiratory syndrome-CoV
MiPEP: miRNA-encoded peptide
miRNAs: MicroRNAs
mRNA: Messenger RNA
NSP3: Nonstructural protein 3
PDCD1: Programmed cell death protein 1
RBD: Receptor-binding domain
Ribozyme: Ribonucleic acid enzyme
RISC: RNA-induced silencing complex
RT-PCR: Real-time polymerase chain reaction
SARS-CoV: Severe acute respiratory syndrome-CoV
TGF: Transforming growth factor
TLR: Toll-like receptors
TMPRSS2: Transmembrane protease serine 2
TNF-*α*: Tumor necrosis factor-alpha
Treg: T regulatory cell
UTR: Untranslated region.
